# Retention in care and viral suppression in the PMTCT continuum at a large referral facility in western Kenya

**DOI:** 10.1007/s10461-022-03666-w

**Published:** 2022-04-25

**Authors:** John M. Humphrey, Julia Songok, Susan Ofner, Beverly Musick, Marsha Alera, Bett Kipchumba, Megan S. McHenry, James G. Carlucci, Jun Park, Winfred Mwangi, Constantin Yiannoutsos, Giorgos Bakoyannis, Kara Wools-Kaloustian

**Affiliations:** 1 Department of Medicine, Indiana University School of Medicine, Indianapolis, IN USA; 2 Department of Paediatrics, Moi University College of Health Sciences, Eldoret, Kenya; 3 Department of Biostatistics, Indiana University School of Medicine, Indianapolis, IN USA; 4 Academic Model Providing Access to Healthcare, Eldoret, Kenya; 5 Department of Reproductive Health, Moi Teaching and Referral Hospital, Eldoret, Kenya; 6 Department of Pediatrics, Indiana University School of Medicine, Indianapolis, IN USA; 7 Department of Biostatistics, Richard M. Fairbanks School of Public Health, Indianapolis, IN USA

**Keywords:** HIV, Pregnant women, Mother-to-child transmission, Retention, Viremia

## Abstract

Medical records of pregnant and postpartum women living with HIV and their infants attending a large referral facility in Kenya from 2015 to 2019 were analyzed to identify characteristics associated with retention in care and viral suppression. Women were stratified based on the timing of HIV care enrollment: known HIV-positive (KHP; enrolled pre-pregnancy) and newly HIV-positive (NHP; enrolled during pregnancy). Associations with retention at 18 months postpartum and viral suppression (< 1000 copies/mL) were determined. Among 856 women (20% NHP), retention was 83% for KHPs and 53% for NHPs. Viral suppression was 88% for KHPs and 93% for NHPs, but 19% of women were missing viral load results. In a competing risk model, viral suppression increased by 18% for each additional year of age but was not associated with other factors. Overall, 1.9% of 698 infants with ≥ 1 HIV test result were HIV-positive. Tailored interventions are needed to promote retention and viral load testing, particularly for NHPs, in the PMTCT continuum.

**Supplementary Information:**

The online version contains supplementary material available at 10.1007/s10461-022-03666-w.

## Introduction

In the prevention of mother-to-child transmission of HIV (PMTCT) cascade in Eastern and Southern Africa, the global regions most affected by HIV, an estimated 95% of pregnant women living with HIV (WLHIV) accessed antiretroviral therapy (ART) for PMTCT in 2019 (1). Despite this achievement, maintaining retention in care and viral suppression remain major challenges for this population. Multiple studies have estimated that nearly a third of women initiating ART during pregnancy become lost to follow-up (LTFU) during the subsequent year, and 30–40% of women retained in care do not maintain viral suppression in the postpartum period (2–7). Viral non-suppression for women living with HIV during pregnancy and the postpartum period is associated with multiple risks, including increased maternal morbidity and mortality, vertical HIV transmission to infants, horizontal transmission to partners, and development and transmission of HIV drug resistance (8–19). In Eastern Africa, for example, an estimated 10,000 of 26,000 new HIV infections in children in 2018 were the result of women not being retained in care and on ART throughout pregnancy and breastfeeding (20). Understanding retention and viral suppression in the PMTCT continuum, and the factors that influence these outcomes, is critical to tailoring effective interventions to this population.

To date, a variety of factors influencing retention and viral suppression in the PMTCT continuum have been identified. Among them, diagnosis of HIV and ART initiation during pregnancy is a pervasive theme, with one study finding that women who initiated ART during pregnancy were five times more likely to disengage from care compared to women who initiated ART because of advanced HIV disease (3, 21). Younger age, HIV-related stigma, fear of disclosure, and lack of social support have also been associated with lower rates of retention and viral suppression (2). Nevertheless, most studies have focused on retention during pregnancy or up to 12 months postpartum, despite data suggesting continued attrition beyond this period (3, 7, 22–29). These studies are also predominantly cross-sectional. Longitudinal measures of retention in the PMTCT continuum have been less frequently reported. In addition, due to feeding practices in Eastern and Southern Africa, breastfeeding and the risk of vertical transmission can last ≥ 18 months (30–33). To further complicate interpretation, many studies were conducted prior to the ‘Treat All’ era of universal ART eligibility, which has contributed to an increasing proportion of WLHIV enrolled in PMTCT services who initiated ART prior to pregnancy (34, 35). Lastly, few studies have examined retention and viral suppression at settings in which maternal and child health services have integrated HIV care, an increasing trend in sub-Saharan Africa that may have a distinct impact on retention and viral suppression compared to non-integrated services (36–39).

Kenya has one of the largest HIV burdens in the world with an HIV prevalence of 6.1% among women of reproductive age (40). Kenya implemented the World Health Organization Option B + policy recommending lifelong ART for all pregnant WLHIV in 2014. This policy was subsumed by the ‘Treat All’ policy in 2016 (41, 42). These policies have facilitated significant reductions in vertical transmission in Kenya, from 26% to 2009 to 11% in 2018 (43–45). Still, PMTCT program-level data on retention and viral suppression in the ‘Treat All’ era are limited. The objective of this study was to determine retention and viral suppression for WLHIV and their infants at a large referral facility in Kenya providing integrated HIV and maternal and child health services, and identify characteristics of WLHIV that may predict poor retention and viral suppression in the ‘Treat All’ era.

## Methods

### Study design

This retrospective cohort study used electronic medical record data from WLHIV who received HIV care during pregnancy at Moi Teaching and Referral Hospital (MTRH) in western Kenya, from 2015 to 2019. The study was approved by the Institutional Research and Ethics Committee at Moi University and MTRH in Kenya. A waiver of informed consent was obtained because the data were routinely collected clinical data that were de-identified prior to analysis. The study was also exempt from IRB review at Indiana University in the United States because the data were de-identified prior to analysis. The reporting of this study follows the STROBE guidelines (see Supplementary Materials) (46).

### Study setting and population

MTRH is the largest public referral hospital in western Kenya. Within MTRH are antenatal clinics (ANC) and postnatal clinics (PNC) that offer integrated HIV services provided by dedicated nurses, clinical officers, and peer counsellors (i.e., Mentor Mothers). Routine viral load testing is also available on site. MTRH is the headquarters of the Academic Model Providing Access to Healthcare (AMPATH). AMPATH is a USAID-funded HIV care and treatment program and a participating site in the International Epidemiology Databases to Evaluate AIDS East Africa consortium (47, 48). Since 2001, AMPATH has enrolled over 250,000 patients living with HIV at Ministry of Health facilities across western Kenya (49, 50). All AMPATH facilities, including MTRH, provide standard-of-care HIV treatment services based on national guidelines (41).

WLHIV were included in the study if they were pregnant or postpartum on or after September 1, 2015 (study start date), had one or more HIV or integrated ANC visits at MTRH during pregnancy, and had a delivery date (or estimated delivery date [EDD] if delivery date was not available) that occurred at least 18 months prior to March 6, 2019 (database closure). All HIV-exposed infants born to eligible WLHIV were also included.

At the time of the study, WHO and Kenyan HIV treatment guidelines recommended efavirenz-based first-line ART for adults ≥ 15 years of age, including women of childbearing age (34, 42). Second-line ART was protease inhibitor (PI) based, either lopinavir or atazanavir, each in combination with ritonavir. For all WLHIV, a viral load was recommended at six months after ART initiation, and if < 1,000 copies/mL, repeated every six months for women who were pregnant or breastfeeding. For any woman with a viral load ≥ 1,000 copies/mL, a viral load was recommended to be repeated after at least three months of enhanced ART adherence counselling and support (41). The transition from first- to second-line ART was based on clinical treatment failure, defined as a viral load ≥ 1,000 copies/mL after ≥ 6 months on ART and ≥ 3 months of excellent adherence, and after addressing any reasons for poor adherence. Typically, AMPATH clients are given an ART quantity that corresponds with the duration of time until their next scheduled appointment, in monthly increments (i.e., a 30-, 60- or 90-day supply). Some pregnant women are given a limited surplus of ART during the month leading up to delivery, to help ensure that they do not have a gap in treatment during the peripartum and early breastfeeding periods. HIV-exposed infants were recommended to have HIV DNA PCR testing at 6 weeks, 6 months, and 12 months, followed by HIV antibody testing at 18 months, which should occur 6 weeks after cessation of breastfeeding.

### Data management

We used clinical and viral load data available in the AMPATH electronic medical record that was collected by clinicians during routine care, initially on paper forms which were back-entered into the electronic medical record by a data clerk, and from 2019 by point-of-care data entry (51).

### Data analysis

Eligible women were delineated based on the timing of enrollment in HIV care: newly HIV-positive (NHP; women enrolled in HIV care during pregnancy) and known HIV-positive (KHP; women enrolled in HIV care before pregnancy). Patient-level, independent variables at the time of enrollment in ANC or pregnancy identification included: age, gestational age, time on ART, antiretroviral base class, viral load (with window of 6 months before or after), CD4 at ART initiation (closest within 90 days before and 7 days after ART initiation), and WHO stage at ART initiation. For the latter, the maximum WHO stage prior to ART initiation was taken to be the WHO stage at ART initiation. If the WHO stage at ART initiation was missing and the next available WHO stage was 1 or 2, then the WHO stage at ART initiation was assumed to be stage 1 or 2. Otherwise, the WHO stage at ART initiation was recorded as missing.

Independent variables following enrollment through 18 months postpartum included: age at delivery, time from ART initiation to delivery, delivery method, pregnancy outcome, duration of breastfeeding, tuberculosis treatment, repeat pregnancy, and viral suppression (defined as < 1,000 copies/mL during pregnancy plus 2 weeks post-delivery and ≥ 6 months post-ART initiation) (42, 52). Percentages of categorical variables were compared between NHPs and KHPs using Chi-square or Fisher’s exact tests. Medians of continuous variables were compared using the Wilcoxon rank sum test.

Retention in care was defined as attending clinic +/- 90 days of four time points: delivery, and 6, 12 and 18 months postpartum. These timepoints align with the infant HIV testing schedule in Kenya and are frequently used as retention endpoints in the PMTCT literature (2, 53). This approach provides a point prevalence of the proportion of women in care at each time point but does not account for patient churn, the movement of women in and out of care, which may occur between these timepoints (54). Therefore, we also calculated the prevalence of gaps in care, defined as missing any scheduled visit by > 30 days, within periods delineated by the above time points. This approach allows for a more in-depth analysis of retention during PMTCT follow-up.

Women who transferred out or died were also enumerated. A threshold of 30 days was selected based on the observed timing of scheduled return visits at the clinic (next paragraph). To analyze the prevalence of gaps in each period, subjects could transition from the ‘gap’ to ‘no gap’ states (or vice versa) across periods, based on the absence or presence of a gap in care, respectively. Women who died or transferred out were not included in subsequent time periods, and women who became LTFU, defined as missing the last scheduled visit by > 30 days and not returning by 18 months postpartum, remained in the ‘gap’ state.

To understand the retention data in the context of routine care at MTRH, we determined the timing and frequency of routine clinic visits to MTRH during pregnancy and the postpartum periods. We calculated the median number of attended visits during these periods, where HIV services were accessed (i.e., HIV clinic or integrated ANC), and the timing of scheduled return visits during PMTCT follow-up. We also calculated the weeks post-delivery at the first postpartum visit, censoring at 90 days postpartum for women who did not have any postpartum visit unless they died or transferred out beforehand. NHPs and KHPs were compared using the log rank test.

Logistic regression models were used to calculate unadjusted and adjusted odds ratios (aOR) and 95% confidence intervals (CI) for independent variables to assess their associations with non-retention (i.e., the inverse of retention defined above) and gaps in care, respectively. Independent variables of age and gestational age were measured at the time of ANC enrollment or pregnancy identification. Other independent variables included WHO stage prior to ART initiation, NHP status and ART base class. The gaps-in-care analysis was set up similar to the non-retention analysis, with the exception that the dependent variable was defined as the occurrence of a gap in care at any point in time from pregnancy to 18 months postpartum.

To examine factors associated with viral suppression during pregnancy, we selected for analysis the subset of women who were KHP, ≥ 6 months post-ART initiation, and had at least one viral load within the period from pregnancy through two weeks post-delivery. If two or more viral load measures were available within this period, the viral load closest to the delivery date was selected. This analysis excluded women who were NHP because Kenya HIV treatment guidelines recommend that an initial viral load test be performed six months after ART initiation (53). Viral suppression at any point during the postpartum period (pregnancy plus two weeks after pregnancy through to 18 months) was modeled using logistic regression. These analyses were conducted using SAS software (55).

Finally, we used a semiparametric proportional odds regression approach for interval-censored competing risk data to analyze the cumulative incidence of viral suppression from two weeks to 18 months post-delivery (censoring event), and ≥ 6 months post-ART initiation (56). This approach was used because the time from delivery to viral suppression was not precisely observed and was only known to lie between the last positive and the first negative viral load (interval censoring), and because losses to follow-up and deaths were competing events to viral suppression. In this analysis, time zero was the date of delivery and 18 months postpartum was the censoring date; LTFU was defined as missing the last scheduled visit by > 30 days and not returning by 18 months postpartum; women who transferred out were censored at the date of the transfer; and, if two or more viral load measures were available within this period, the earliest chronologic viral load was used for analysis. Women who became LTFU were conservatively assumed to be not virally suppressed at the time that they became LTFU. The covariates used in both of these models were the same as those used in the logistic models of non-retention and gaps in care described above. Time on ART was not included in this model due to its collinearity with NHP status. Women with no visits following delivery were excluded. This analysis was performed using the function ciregic in the R package intccr (57).

## Results

### Characteristics of women at enrollment in ANC or pregnancy identification

A total of 967 pregnant and postpartum WLHIV enrolled in care on or after September 1, 2015 and had a delivery date that occurred at least 18 months prior to database closure. Of these, 111 were excluded because they had no HIV or integrated ANC visits at MTRH during pregnancy, resulting in 856 pregnant WLHIV eligible for analysis along with their HIV-exposed infants. Among the eligible women, 167 (20%) were NHPs and 689 (80%) were KHPs (Table 1). Compared to KHPs, NHPs had a lower median age and greater gestational age at pregnancy identification, higher proportion with WHO stage 1 or 2, and higher median CD4 at ART initiation. Among 544 KHPs with a viral load +/- 6 months of pregnancy identification, 87% were suppressed.

**Table 1:** Characteristics of women at enrollment in ANC or pregnancy identification, delineated by timing of HIV diagnosis

Characteristic	Total n (%) n = 856	New HIV-Positive n (%) n = 167	Known HIV-Positive n (%) n = 689	Test Statistic	P-value^a^
Age, median years (IQR)	31 (27–36)	28 (23–32)	32 (28–36)	Z = -8.0	< 0.001
Gestation age, median weeks (IQR)	20 (14–28)	24 (17–31)	20 (13–27)	Z = 4.5	< 0.001
Maximum WHO stage prior to ART initiation					
WHO stage 1 or 2	660 (77)	156 (93)	504 (73)	X^2^ = 41.5	< 0.001
WHO stage 3 or 4	169 (20)	3 (1.8)	166 (24)		
Missing	27 (3.2)	8 (4.8)	19 (2.8)		
CD4 at ART initiation, median (IQR)^b^	233 (139–390)	365 (204–572)	226 (136–362)	Z = 3.8	< 0.001
Time on ART, median years (IQR)	2.7 (0.1–5.6)	0.0 (0.0–0.0)	3.8 (1.5–6.3)	Z = -18.3	< 0.001
ART base class					
NNRTI	763 (89)	163 (98)	600 (87)	n/a^c^	< 0.001
PI	87 (10)	1 (0.6)	86 (13)		
Missing	6 (0.7)	3 (1.8)	3 (0.4)		
Viral load suppressed					
Yes	471 (55)	n/a	471 (68)		n/a
No	73 (8.5)	n/a	73 (11)		
Missing	312 (36)	n/a	145 (21)		

IQR, interquartile range; NNRTI, non-nucleoside reverse transcriptase inhibitor; PI, protease inhibitor

^a^ Calculated differences between groups exclude those with missing data

^b^ CD4 available for 395 KHPs and 54 NHPs

^c^ Fisher’s Exact Test. Test statistic not available

### Characteristics of women through 18 months postpartum

The median time from ART initiation to delivery for NHPs and KHPs was 15 and 218 weeks, respectively (Table 2). The pregnancy outcome was documented for 87% of women. Miscarriages and pre-term deliveries were each documented in < 1% of pregnancies. A total of 361 (52%) women had a documented viral load within the 12 months prior to pregnancy, and among them, 88% were virally suppressed. During pregnancy, viral suppression among 404 KHPs with an available viral load was 88%. Viral suppression among NHPs and KHPs with a postpartum viral load was 93% and 88%, respectively. However, 41% of NHPs and 13% of KHPs did not have a documented postpartum viral load. This was partly due to attrition, as 42% of NHPs and 29% of KHPs missing a viral load had either died or become LTFU before a viral load could be obtained.

**Table 2:** Characteristics of women and their infants through 18 months postpartum, delineated by timing of HIV diagnosis

Characteristic	Total n (%) n = 856	New HIV-Positive n (%) n = 167	Known HIV-Positive n (%) n = 689	Test Statistic	P-value^a^
Age at delivery, median years (IQR)	32 (27–36)	28 (24–32)	33 (29–37)	Z = -8.1	< 0.001
Time from ART initiation to delivery, median weeks (IQR)	155 (30–310)	15 (8–23)	218 (98–344)	Z = -18.4	< 0.001
Pregnancy outcome					
Miscarriage	5 (0.6)	1 (0.6)	4 (0.6)	n/a^f^	0.999
Pre-term delivery	4 (0.5)	0 (0)	4 (0.6)		
Term delivery	740 (86)	126 (75)	614 (89)		
Missing	107 (13)	40 (24)	67 (9.7)		
Method of delivery^b^					
Vaginal	604 (81)	99 (79)	505 (81)	X^2^ = 0.1	0.757
C-section	108 (15)	19 (15)	89 (14)		
Missing	34 (4.6)	7 (5.6)	27 (4.3)		
Tuberculosis treatment	16 (1.9)	2 (1.2)	14 (2.0)	n/a^f^	0.750
Repeat pregnancy	25 (2.9)	2 (1.2)	23 (3.3)	n/a^f^	0.199
Viral load suppressed in pregnancy					
Yes	360 (42)	4 (2.4)	356 (52)	X^2^ = 0.5	0.463
No	48 (5.6)	0 (0)	48 (7.0)		
Missing	448 (52)	163 (98)	285 (41)		
Viral load suppressed postpartum^c^					
Yes	615 (72)	91 (55)	524 (76)	X^2^ = 2.3	0.126
No	82 (9.6)	7 (4.2)	75 (11)		
Missing	159 (19)	69 (41)	90 (13)		
Duration of breastfeeding, median weeks (IQR)^d^	48 (38–54)	47 (28–53)	48 (39–54)	Z = -2.0	0.049
Duration of exclusive breastfeeding, median weeks (IQR)^e^	26 (20–28)	26 (20–28)	26 (20–28)	Z = 0.0	0.982
Postpartum retention in care					
6 months	749 (88)	116 (70)	633 (92)	X^2^ = 61.7	< 0.001
12 months	727 (85)	112 (67)	615 (89)	X^2^ = 51.7	< 0.001
18 months	661 (77)	88 (53)	573 (83)	X^2^ = 70.9	< 0.001
LTFU	229 (27)	69 (41)	160 (23)	X^2^ = 22.5	< 0.001
Death	4 (0.5)	1 (0.6)	3 (0.4)	n/a^f^	0.581

^a^ Calculated differences between groups exclude those with missing data

^b^ N = 746 (NHP: 125, KHP: 621) because these data are only captured in the infant’s data collection form

^c^ Number of viral loads available for each subject are 0 viral loads (n = 151 subjects), 1 (n = 263), 2 (n = 330), 3–7 (n = 112)

^d^ N = 720 (NHP:121, KHP:599)

^e^ N = 602 (NHP:103, KHP:499)

^f^ Fisher’s Exact Test. Test statistic not available

**Fig. 1:**
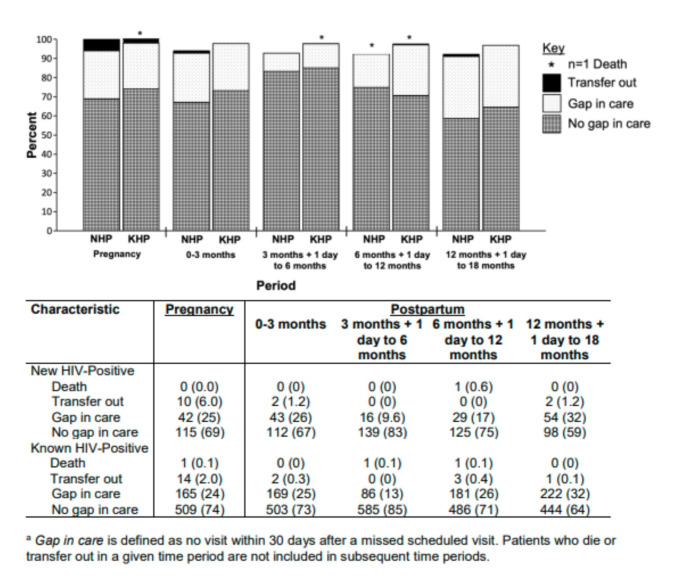
Gaps in care during pregnancy and the postpartum period (n = 856).^a^

Documented mortality was low, occurring for one NHP and three KHPs (Fig. 1). Transfers out were also low, occurring for 2.5% of NHPs and 0.9% of KHPs during the postpartum period. During pregnancy, 25% of NHPs and 24% of KHPs experienced a gap in care. The prevalence of gaps in care ranged from 10 to 32% across postpartum periods and was highest during the period from 12 to 18 months postpartum. Overall retention at 6 months postpartum was 88%, decreasing to 85% and 77% at 12 and 18 months, respectively (Table 2). A higher proportion of KHPs compared to NHPs were retained at each time point, with 83% of KHPs and 53% of NHPs retained at 18 months postpartum. Cumulatively, 41% of NHPs and 23% of KHPs became LTFU by 18 months postpartum. The routine postpartum visit schedule pattern that emerged indicated that monthly return visits were scheduled for the first 12 months postpartum followed by return visits scheduled every two months starting in month 13 (Table S1).

There was no difference in the duration of breastfeeding among infants born to NHPs versus KHPs. The median durations of any and exclusive breastfeeding among all infants were 48 and 26 weeks, respectively. Overall, 89% of women retained in care were still breastfeeding at 6 months postpartum, 39% at 12 months, and 1.5% at 18 months.

### Characteristics of infants

Among 698 infants with one or more HIV test results from birth through 18 months postpartum, 1.9% were HIV positive (Table S2). However, 40–65% of all infants known to be alive did not have a documented HIV test result at each of the 6 week and 6, 12 and 18 month time points. Among infants retained in care at 18 months, 95% had stopped breastfeeding and of these, 39% were missing an HIV test after stopping breastfeeding.

### Associations with gaps in care and non-retention

Older age was associated with a slightly lower adjusted odds of experiencing a gap in care (aOR = 0.96; Table 3). Older age was also associated with a lower adjusted odds of non-retention at 18 months (aOR = 0.95). Other factors associated with a higher adjusted odds of non-retention include receipt of a PI/other ART base class (aOR = 1.88) and NHP status (aOR = 3.79),

**Table 3:** Factors associated with experiencing a gap in care and non-retention in care, respectively

	Gap in care n = 792^a^		Non-retention at 18 months n = 829^b^	
**Characteristic**	**Unadjusted OR** **(95% CI)**	**Adjusted OR** **(95% CI)**	**Unadjusted OR** **(95% CI)**	**Adjusted OR** **(95% CI)**
Age (years)	0.97 (0.94–0.99)	0.96 (0.93–0.99)	0.93 (0.90–0.95)	0.95 (0.92–0.97)
Gestational age (weeks)	0.98 (0.96-1.00)	0.98 (0.96-1.00)	0.99 (0.98–1.01)	0.98 (0.96-1.00)
Maximum WHO stage pre-ART initiation				
WHO stage 1 or 2	Ref.	Ref.	Ref.	Ref.
WHO stage 3 or 4	1.41 (0.87–2.26)	1.51 (0.92–2.47)	0.51 (0.32–0.82)	0.78 (0.47–1.28)
ART base class				
NNRTI	Ref.	Ref.	Ref.	Ref.
PI/other	1.39 (0.72–2.71)	1.41 (0.72–2.78)	1.18 (0.69–2.01)	1.88 (1.07–3.30)
NHP status	0.94 (0.60–1.49)	0.99 (0.60–1.63)	4.23 (2.92–6.14)	3.79 (2.50–5.76)

^a^ Includes 646 with gap and 146 without gap

^b^ Includes 644 retained and 185 not retained women

### Associations with viral suppression during pregnancy

In the adjusted logistic regression model, KHPs with documented pre-pregnancy viral suppression had four times the odds of being virally suppressed during pregnancy compared to KHPs without pre-pregnancy viral suppression (aOR = 4.05; Table S3). Older age was associated with a slightly higher adjusted odds of viral suppression (aOR = 1.10). Those with WHO stages 3 or 4 pre-ART initiation had lower adjusted odds of being virally suppressed during pregnancy compared to those with WHO stages 1 or 2 (aOR = 0.45).

### Associations with viral suppression following delivery

In the competing risk model for time to viral suppression following delivery, the adjusted odds of viral suppression increased by 18% for each additional year of age at delivery (Table 4; see Table S4 for summary statistics by event). Conversely, the adjusted odds of death/LTFU decreased by 13% for each additional year of age at delivery. NHP status was not associated with viral suppression or death/LTFU. In a sensitivity analysis including only subjects with one or more available postpartum viral loads, the estimates from the bivariate models were similar to those in the entire cohort (Table S5).

**Table 4:** Competing risk model for factors associated with time to viral suppression following delivery

	Model of viral suppression ^a^		Model of death/LTFU ^a^	
**Characteristic**	**Unadjusted OR** **(95% CI)**	**Adjusted OR** **(95% CI)**	**Unadjusted OR** **(95% CI)**	**Adjusted OR** **(95% CI)**
Age (years)	1.15 (1.13–1.17)	1.18 (1.12–1.25)	0.89 (0.87–0.91)	0.87 (0.82–0.93)
Gestational age (weeks)	1.10 (1.07–1.12)	0.99 (0.94–1.03)	0.94 (0.92–0.96)	1.03 (0.97–1.08)
Maximum WHO stage prior to ART initiation				
WHO stage 1 or 2	Ref.	Ref.	Ref.	Ref.
WHO stage 3 or 4	2.56 (1.91–3.43)	0.92 (0.72–1.18)	1.22 (0.73–2.03)	1.16 (0.78–1.74)
ART base class				
NNRTI	Ref.	Ref.	Ref.	Ref.
PI/other	2.37 (1.75–3.20)	0.94 (0.70–1.27)	2.36 (1.40–3.97)	1.10 (0.72–1.68)
NHP status	2.73 (2.07–3.58)	1.18 (0.93–1.49)	1.41 (0.90–2.22)	1.36 (0.85–2.17)

^a^ Includes 770 women with complete data

## Discussion

In this study, we found that NHPs had four times the odds of not being retained in care by 18 months postpartum compared to KHPs after controlling for other factors. This finding is consistent with those of prior studies and emphasizes that a new HIV diagnosis during pregnancy is a significant risk factor for non-retention in the PMTCT continuum (3, 21). KHPs who initiated ART under the ‘Treat All’ policy had likely already negotiated the barriers to retention and ART adherence that a new HIV diagnosis presents, and therefore had lower odds of disengaging from care compared to NHPs. Overall retention at 12 months postpartum in our study was 67% for NHPs and 89% for KHPs. These rates are similar to those reported in a study by Akama et al in western Kenya, which found that retention was 66% for NHPs and 82% for KHPs by approximately 12 months postpartum (21). In our study, approximately a third of women who became LTFU did so after 12 months postpartum, however, resulting in lower retention at 18 months (53% for NHPs and 83% for KHPs) compared to at 12 months. This demonstrates the importance of examining retention throughout the entire PMTCT continuum. It is clear that retention in care remains a formidable challenge at this integrated, public-sector PMTCT program in the ‘Treat All’ era. Addressing this challenge will require enhanced service delivery to NHPs who are at highest risk of non-retention in the PMTCT continuum. Intensified mentor mother support, disclosure counselling, male partner involvement, text messaging, and conditional cash transfers may be scalable and economically feasible interventions to enhance service delivery for this population (58–65).

Gaps in care were also prevalent, occurring for more than 20% of women during pregnancy and 10–32% of women during the postpartum periods. These gaps could indicate ART interruptions and subsequent HIV viremia, increasing the risks of onward HIV transmission and development of drug resistance. For the program, these gaps could also indicate significant consumption of its patient outreach and defaulter tracking resources to prevent women from becoming LTFU. Alternatively, these gaps could represent mostly benign variations in appointment adherence among women with adequate quantities of ART, who are simply adjusting their clinic schedule to accommodate their work and other responsibilities. In this scenario, patient outreach and tracking are not needed. In a multiple regression analysis, age was the only factor that discriminated modestly between women who did and did not have a gap in care. Thus, while non-retention defined cross-sectionally at 18 months postpartum could largely be predicted by NHP status, gaps in care could not. Further research is needed to understand the etiologies and clinical impact of gaps in PMTCT care to determine when and how programs should optimally intervene when they occur.

We also found that postpartum viral suppression was 88%, approaching the UNAIDS final ‘90’ target of ≥ 90% viral suppression among those on ART (66). However, this finding primarily pertains to women who were retained in care and had a viral load test. In our study, 41% of NHPs and 13% of KHPs were missing a postpartum viral load test, which was partly due to attrition. Viral suppression was likely lower among postpartum WLHIV who had become LTFU (67). Moreover, 29% of NHPs and 46% of KHPs who were missing a VL did so despite being in care at a facility with on-site viral load testing. This is a significant shortcoming that must be addressed to achieve the final ‘90’ for this population. Continuous quality improvement approaches could be adopted to overcome barriers to viral load testing and adherence to testing guidelines (23, 68, 69). These approaches could also be used to improve uptake of HIV testing for infants, among whom a high proportion were missing HIV test results in our study (70–72).

Finally, only older age, and not NHP status, was associated with time to viral suppression following delivery. This finding suggests that NHPs who are able to overcome the challenge of being diagnosed with HIV during pregnancy and remain in care are also able to achieve viral suppression comparable to KHPs. A similar selection effect probably also explains the finding in our study that pre-pregnancy viral suppression among KHPs is a significant predictor of viral suppression during pregnancy (73). While the majority of postpartum viremic episodes among women initiating ART during pregnancy has been attributed to poor adherence (18, 74), postpartum viremia among KHPs is more likely to be associated with virologic failure and emergence of drug resistance (75). Thus, enhancing postpartum viral suppression may still require interventions specifically designed for each group.

Our study has strengths and limitations. Strengths include its large sample size and use of routine program data likely reflecting typical care environments in the region. Our study also addresses knowledge gaps concerning retention and viral suppression in the PMTCT continuum in the ‘Treat All’ era, and in the context of a facility providing integrated HIV and maternal and child health services. The breadth the AMPATH program, whose catchment covers much of western Kenya, is also a strength, as it facilitates verification that pregnant women newly presenting to care are indeed NHP rather than silently transferring in or re-engaging in care after a period of disengagement. Limitations include the use of retrospective data, which limited our ability to understand the social-ecological factors driving non-retention or viral non-suppression for WLHIV (e.g. stigma and disclosure, mental health, and socioeconomic status). Missing data, particularly maternal viral loads and infant HIV tests, may also limit extrapolation of our findings to broader populations. For example, missing viral load and HIV test results may indicate missed appointments, potentially implying that patients with test results had higher levels of engagement in care and ART adherence compared to those without test results. Missing viral loads may also be more prevalent in patients with a history of poor ART adherence, as clinicians may have deferred viral load testing until their adherence was optimized. The proportion of preterm births was also lower than expected in our retrospective cohort at 0.5%, compared to 10–20% among prospective cohorts in the region (76, 77). It is likely that preterm births are enriched among the 13% of women whose delivery outcomes were missing (Table 2). The definition of retention used in this study was also conservative. More restrictive definitions would likely have yielded even lower retention rates. Two significant healthcare worker strikes also occurred in Kenya in 2017. These events affected public sector health facilities throughout the country, potentially hindering women’s ability to access PMTCT services and maintain retention in care (78).

## Conclusions

Retention in care during the ‘Treat All’ era was suboptimal at a large referral facility in Kenya providing integrated HIV and maternal and child health services. Retention among NHPs was substantially lower than retention among KHPs, underscoring the need for interventions tailored to this vulnerable population.

### Disclaimer

The content is solely the responsibility of the authors and does not necessarily represent the official views of the National Institutes of Health.

**List of abbreviations**.

AMPATH, Academic Model Providing Access to Healthcare; ANC, antenatal clinic; aOR, adjusted Odds Ratio; ART, antiretroviral therapy; ARV, antiretroviral; CI, confidence interval; IQR, interquartile range; KHP; Known HIV-positive; MTRH, Moi Teaching and Referral Hospital; NHP; Newly HIV-positive; PNC, postnatal clinic; WHO, World Health Organization; WLHIV, women living with HIV.

## Electronic Supplementary Material

Below is the link to the electronic supplementary material.

Supplementary Material 1

Supplementary Material 2

## Data Availability

The data analyzed during the study are available from the corresponding author on reasonable request.

## References

[1] Joint United Nations Programme on HIV/AIDS. UNAIDS Data. 2020. Geneva: UNAIDS; 2020. Available from: https://www.unaids.org/sites/default/files/media_asset/2020_aids-data-book_en.pdf. Accessed July 21, 2020.

[2] Knettel BA, Cichowitz C, Ngocho JS, Knippler ET, Chumba LN, Mmbaga BT (2018). Retention in HIV Care During Pregnancy and the Postpartum Period in the Option B + Era: Systematic Review and Meta-Analysis of Studies in Africa. J Acquir Immune Defic Syndr.

[3] Tenthani L, Haas AD, Tweya H, Jahn A, van Oosterhout JJ, Chimbwandira F (2014). Retention in care under universal antiretroviral therapy for HIV-infected pregnant and breastfeeding women (‘Option B+’) in Malawi. AIDS.

[4] Clouse K, Vermund SH, Maskew M, Lurie MN, MacLeod W, Malete G (2017). Mobility and Clinic Switching Among Postpartum Women Considered Lost to HIV Care in South Africa. J Acquir Immune Defic Syndr.

[5] Joseph J, Gotora T, Erlwanger AS, Mushavi A, Zizhou S, Masuka N (2017). Impact of Point-of-Care CD4 Testing on Retention in Care Among HIV-Positive Pregnant and Breastfeeding Women in the Context of Option B + in Zimbabwe: A Cluster Randomized Controlled Trial. J Acquir Immune Defic Syndr.

[6] Phillips TK, Clouse K, Zerbe A, Orrell C, Abrams EJ, Myer L (2018). Linkage to care, mobility and retention of HIV-positive postpartum women in antiretroviral therapy services in South Africa. J Int AIDS Soc.

[7] Haas AD, Tenthani L, Msukwa MT, Tal K, Jahn A, Gadabu OJ (2016). Retention in care during the first 3 years of antiretroviral therapy for women in Malawi’s option B + programme: an observational cohort study. Lancet HIV.

[8] Myer L, Dunning L, Lesosky M, Hsiao NY, Phillips T, Petro G (2017). Frequency of Viremic Episodes in HIV-Infected Women Initiating Antiretroviral Therapy During Pregnancy: A Cohort Study. Clin Infect Dis.

[9] Sam-Agudu NA, Ramadhani HO, Isah C, Anaba U, Erekaha S, Fan-Osuala C (2017). The Impact of Structured Mentor Mother Programs on 6-Month Postpartum Retention and Viral Suppression among HIV-Positive Women in Rural Nigeria: A Prospective Paired Cohort Study. J Acquir Immune Defic Syndr.

[10] Langwenya N, Phillips TK, Brittain K, Zerbe A, Abrams EJ, Myer L (2018). Same-day antiretroviral therapy (ART) initiation in pregnancy is not associated with viral suppression or engagement in care: A cohort study. J Int AIDS Soc.

[11] Chetty T, Newell ML, Thorne C, Coutsoudis A (2018). Viraemia before, during and after pregnancy in HIV-infected women on antiretroviral therapy in rural KwaZulu-Natal, South Africa, 2010–2015. Trop Med Int Health.

[12] Maartens G, Celum C, Lewin SR (2014). HIV infection: epidemiology, pathogenesis, treatment, and prevention. Lancet.

[13] Rousseau CM, Nduati RW, Richardson BA, Steele MS, John-Stewart GC, Mbori-Ngacha DA (2003). Longitudinal analysis of human immunodeficiency virus type 1 RNA in breast milk and of its relationship to infant infection and maternal disease. J Infect Dis.

[14] Mmiro FA, Aizire J, Mwatha AK, Eshleman SH, Donnell D, Fowler MG (2009). Predictors of early and late mother-to-child transmission of HIV in a breastfeeding population: HIV Network for Prevention Trials 012 experience, Kampala, Uganda. J Acquir Immune Defic Syndr.

[15] Davis NL, Miller WC, Hudgens MG, Chasela CS, Sichali D, Kayira D (2016). Maternal and Breastmilk Viral Load: Impacts of Adherence on Peripartum HIV Infections Averted-The Breastfeeding, Antiretrovirals, and Nutrition Study. J Acquir Immune Defic Syndr.

[16] Mugo NR, Heffron R, Donnell D, Wald A, Were EO, Rees H (2011). Increased risk of HIV-1 transmission in pregnancy: a prospective study among African HIV-1-serodiscordant couples. AIDS.

[17] Joseph Davey D, Farley E, Gomba Y, Coates T, Myer L (2018). Sexual risk during pregnancy and postpartum periods among HIV-infected and -uninfected South African women: Implications for primary and secondary HIV prevention interventions. PLoS ONE.

[18] Hosseinipour M, Nelson JAE, Trapence C, Rutstein SE, Kasende F, Kayoyo V (2017). Viral Suppression and HIV Drug Resistance at 6 Months Among Women in Malawi’s Option B + Program: Results From the PURE Malawi Study. J Acquir Immune Defic Syndr.

[19] Ngarina M, Kilewo C, Karlsson K, Aboud S, Karlsson A, Marrone G (2015). Virologic and immunologic failure, drug resistance and mortality during the first 24 months postpartum among HIV-infected women initiated on antiretroviral therapy for life in the Mitra plus Study, Dar es Salaam, Tanzania. BMC Infect Dis.

[20] Joint United Nations Programme on HIV/AIDS. Free S, Free S, Free AIDS. Geneva: UNAIDS; 2019. Available from: https://free.unaids.org. Accessed July 21, 2020.

[21] Akama E, Nimz A, Blat C, Moghadassi M, Oyaro P, Maloba M (2019). Retention and viral suppression of newly diagnosed and known HIV positive pregnant women on Option B + in Western Kenya. AIDS Care.

[22] Mwapasa V, Joseph J, Tchereni T, Jousset A, Gunda A (2017). Impact of Mother-Infant Pair Clinics and Short-Text Messaging Service (SMS) Reminders on Retention of HIV-Infected Women and HIV-Exposed Infants in eMTCT Care in Malawi: A Cluster Randomized Trial. J Acquir Immune Defic Syndr.

[23] Oyeledun B, Phillips A, Oronsaye F, Alo OD, Shaffer N, Osibo B (2017). The Effect of a Continuous Quality Improvement Intervention on Retention-In-Care at 6 Months Postpartum in a PMTCT Program in Northern Nigeria: Results of a Cluster Randomized Controlled Study. J Acquir Immune Defic Syndr.

[24] Ford D, Muzambi M, Nkhata MJ, Abongomera G, Joseph S, Ndlovu M (2017). Implementation of Antiretroviral Therapy for Life in Pregnant/Breastfeeding HIV + Women (Option B+) Alongside Rollout and Changing Guidelines for ART Initiation in Rural Zimbabwe: The Lablite Project Experience. J Acquir Immune Defic Syndr.

[25] Chan AK, Kanike E, Bedell R, Mayuni I, Manyera R, Mlotha W (2016). Same day HIV diagnosis and antiretroviral therapy initiation affects retention in Option B + prevention of mother-to-child transmission services at antenatal care in Zomba District, Malawi. J Int AIDS Soc.

[26] Dzangare J, Takarinda KC, Harries AD, Tayler-Smith K, Mhangara M, Apollo TM (2016). HIV testing uptake and retention in care of HIV-infected pregnant and breastfeeding women initiated on ‘Option B+’ in rural Zimbabwe. Trop Med Int Health.

[27] Tweya H, Gugsa S, Hosseinipour M, Speight C, Ng’ambi W, Bokosi M (2014). Understanding factors, outcomes and reasons for loss to follow-up among women in Option B + PMTCT programme in Lilongwe, Malawi. Trop Med Int Health.

[28] Mitiku I, Arefayne M, Mesfin Y, Gizaw M (2016). Factors associated with loss to follow-up among women in Option B + PMTCT programme in northeast Ethiopia: a retrospective cohort study. J Int AIDS Soc.

[29] Carlucci JG, Liu Y, Friedman H, Pelayo BE, Robelin K, Sheldon EK, Clouse K, Vermund SH (2018). Attrition of HIV-exposed infants from early infant diagnosis services in low- and middle-income countries: a systematic review and meta-analysis. J Int AIDS Soc.

[30] National Bureau of Statistics-Kenya and ICF International. 2015. 2014 Kenya Demographic and Health Survey Key Findings. Rockville, Maryland, USA. KNBS and ICF International. Available from: https://www.dhsprogram.com/pubs/pdf/sr227/sr227.pdf. Accessed January 12, 2021.

[31] Ogbo FA, Agho K, Ogeleka P, Woolfenden S, Page A, Eastwood J (2017). Infant feeding practices and diarrhoea in sub-Saharan African countries with high diarrhoea mortality. PLoS ONE.

[32] World Health Organization. Guideline: Updates on HIV and infant feeding. Geneva: WHO; 2016. Available from: https://apps.who.int/iris/bitstream/handle/10665/246260/9789241549707-eng.pdf. Accessed January 12, 2021.

[33] Flynn PM, Taha TE, Cababasay M, Fowler MG, Mofenson LM, Owor M (2018). Prevention of HIV-1 Transmission Through Breastfeeding: Efficacy and Safety of Maternal Antiretroviral Therapy Versus Infant Nevirapine Prophylaxis for Duration of Breastfeeding in HIV-1-Infected Women With High CD4 Cell Count (IMPAACT PROMISE): A Randomized, Open-Label, Clinical Trial. J Acquir Immune Defic Syndr.

[34] World Health Organization. Consolidated Guidelines on the Use of Antiretroviral Drugs for Treating and Preventing HIV Infection: Recommendations for a Public Health Approach. Geneva: WHO; 2016. Available from: http://apps.who.int/iris/bitstream/10665/208825/1/9789241549684_eng.pdf. Accessed January 15, 2021. 27466667

[35] Myer L, Phillips TK (2017). Beyond “Option B+”: Understanding Antiretroviral Therapy (ART) Adherence, Retention in Care and Engagement in ART Services Among Pregnant and Postpartum Women Initiating Therapy in Sub-Saharan Africa. J Acquir Immune Defic Syndr.

[36] Turan JM, Bukusi EA, Onono M, Steinfeld R, Washington S, Shade S (2012). Effects of Antenatal Care-HIV Service Integration on the Prevention of Mother-to-Child Transmission Cascade: Results from a cluster-randomized controlled trial in Kenya. Presented at: Integration for Impact.

[37] Geldsetzer P, Yapa HM, Vaikath M, Ogbuoji O, Fox MP, Essajee SM (2016). A systematic review of interventions to improve postpartum retention of women in PMTCT and ART care. J Int AIDS Soc.

[38] Herlihy JM, Hamomba L, Bonawitz R, Goggin CE, Sambambi K, Mwale J (2015). Implementation and Operational Research: Integration of PMTCT and Antenatal Services Improves Combination Antiretroviral Therapy Uptake for HIV-Positive Pregnant Women in Southern Zambia: A Prototype for Option B+?. J Acquir Immune Defic Syndr.

[39] Myer L, Phillips TK, Zerbe A, Brittain K, Lesosky M, Hsiao NY (2018). Integration of postpartum healthcare services for HIV-infected women and their infants in South Africa: A randomised controlled trial. PLoS Med.

[40] Joint United Nations Programme on HIV/AIDS. AIDSinfo. Geneva: UNAIDS; 2020. Available from: http://aidsinfo.unaids.org/. Accessed July 21, 2020.

[41] National AIDS and STI Control Programme. Guidelines on use of antiretroviral drugs for treating and preventing HIV infection: rapid advice. Nairobi: NASCOP; 2014. Available from: http://guidelines.health.go.ke. Accessed July 12, 2020.

[42] National AIDS. and STI Control Programme. Guidelines on Use of Antiretroviral Drugs for Treating and Preventing HIV Infection in Kenya. Nairobi: NASCOP; 2016. Available from: http://guidelines.health.go.ke. Accessed July 12, 2020.

[43] Kenya Ministry of Health. Towards the elimination of mother to child transmission of HIV and keeping mothers alive, 2012–2015. Nairobi: NASCOP; 2012. Available from: http://guidelines.health.go.ke. Accessed July 9, 2020.

[44] Joint United Nations Programme on HIV/AIDS. UNAIDS Data. 2019. Geneva: UNAIDS; 2019. Available from: https://www.unaids.org/en/resources/documents/2019/2019-UNAIDS-data. Accessed March 5, 2020.

[45] National AIDS and STI Control Programme. Kenya HIV, Estimates Report 2018. Nairobi. NASCOP; 2018. Available from: https://nacc.or.ke/wp-content/uploads/2018/11/HIV-estimates-report-Kenya-20182.pdf. Accessed July 6, 2020.

[46] von Elm E, Altman DG, Egger M, Pocock SJ, Gotzsche PC, Vandenbroucke JP (2008). The Strengthening the Reporting of Observational Studies in Epidemiology (STROBE) statement: guidelines for reporting observational studies. J Clin Epidemiol.

[47] Academic Model Providing Access to Healthcare. AMPATH; 2017. Available at: http://www.ampathkenya.org.

[48] Egger M, Ekouevi DK, Williams C, Lyamuya RE, Mukumbi H, Braitstein P (2012). Cohort Profile: the international epidemiological databases to evaluate AIDS (IeDEA) in sub-Saharan Africa. Int J Epidemiol.

[49] Einterz RM, Kimaiyo S, Mengech HN, Khwa-Otsyula BO, Esamai F, Quigley F (2007). Responding to the HIV pandemic: the power of an academic medical partnership. Acad Med.

[50] Wools-Kaloustian K, Kimaiyo S, Musick B, Sidle J, Siika A, Nyandiko W (2009). The impact of the President’s Emergency Plan for AIDS Relief on expansion of HIV care services for adult patients in western Kenya. AIDS.

[51] Tierney WM, Rotich JK, Hannan TJ, Siika AM, Biondich PG, Mamlin BW (2007). The AMPATH medical record system: creating, implementing, and sustaining an electronic medical record system to support HIV/AIDS care in western Kenya. Stud Health Technol Inform.

[52] AIDSinfo. Guidelines for the Use of Antiretroviral Agents in Pediatric HIV Infection. 2016. Available from: https://clinicalinfo.hiv.gov/en/guidelines/pediatric-arv/whats-new-guidelines. Accessed January 5, 2021.

[53] National AIDS. and STI Control Programme. Guidelines on Use of Antiretroviral Drugs for Treating and Preventing HIV Infection in Kenya. Nairobi: NASCOP; 2018. Available from: http://guidelines.health.go.ke. Accessed July 22, 2020.

[54] Gill MJ, Krentz HB (2009). Unappreciated epidemiology: the churn effect in a regional HIV care programme. Int J STD AIDS.

[55] SAS Institute, Inc. Cary NC. 2016. Available from: www.sas.com/sas.

[56] Bakoyannis G, Yu M, Yiannoutsos CT (2017). Semiparametric regression on cumulative incidence function with interval-censored competing risks data. Stat Med.

[57] Park J, Bakoyannis G, Yiannoutsos CT (2019). Semiparametric competing risks regression under interval censoring using the R package intccr. Comput Methods Programs Biomed.

[58] Ambia J, Mandala J (2016). A systematic review of interventions to improve prevention of mother-to-child HIV transmission service delivery and promote retention. J Int AIDS Soc.

[59] Phiri S, Tweya H, van Lettow M, Rosenberg NE, Trapence C, Kapito-Tembo A (2017). Impact of Facility- and Community-Based Peer Support Models on Maternal Uptake and Retention in Malawi’s Option B + HIV Prevention of Mother-to-Child Transmission Program: A 3-Arm Cluster Randomized Controlled Trial (PURE Malawi). J Acquir Immune Defic Syndr.

[60] Sam-Agudu NA, Ramadhani HO, Isah C, Anaba U, Erekaha S, Fan-Osuala C (2017). The Impact of Structured Mentor Mother Programs on 6-Month Postpartum Retention and Viral Suppression among HIV-Positive Women in Rural Nigeria: A Prospective Paired Cohort Study. J Acquir Immune Defic Syndr.

[61] Aliyu MH, Blevins M, Audet CM, Kalish M, Gebi UI, Onwujekwe O (2016). Integrated prevention of mother-to-child HIV transmission services, antiretroviral therapy initiation, and maternal and infant retention in care in rural north-central Nigeria: a cluster-randomised controlled trial. Lancet HIV.

[62] Audet CM, Blevins M, Chire YM, Aliyu MH, Vaz LM, Antonio E (2016). Engagement of Men in Antenatal Care Services: Increased HIV Testing and Treatment Uptake in a Community Participatory Action Program in Mozambique. AIDS Behav.

[63] Takah NF, Kennedy ITR, Johnman C (2017). The impact of approaches in improving male partner involvement in the prevention of mother-to-child transmission of HIV on the uptake of maternal antiretroviral therapy among HIV-seropositive pregnant women in sub-Saharan Africa: a systematic review and meta-analysis. BMJ Open.

[64] Yotebieng M, Thirumurthy H, Moracco KE, Kawende B, Chalachala JL, Wenzi LK (2016). Conditional cash transfers and uptake of and retention in prevention of mother-to-child HIV transmission care: a randomised controlled trial. Lancet HIV.

[65] Sarko KA, Blevins M, Ahonkhai AA, Audet CM, Moon TD, Gebi UI (2017). HIV status disclosure, facility-based delivery and postpartum retention of mothers in a prevention clinical trial in rural Nigeria. Int Health.

[66] Joint United Nations Programme on HIV/AIDS. 90-90-90: an ambitious treatment target to help end the AIDS epidemic. Geneva: UNAIDS; 2014. Available from: https://www.unaids.org/en/resources/909090. Accessed September 5, 2020.

[67] Humphrey J, Musick B, Songok J, Kipchumba B, Kosgei W, Mwangi W, et al. A comparison of the outcomes of women retained versus lost during the prevention of mother to child HIV transmission (PMTCT) cascade: the IeDEA-Kenya PMTCT cohort. AIDS 2020; July 6–10, 2020; San Francisco, CA (virtual).

[68] Yotebieng M, Mpody C, Ravelomanana NL, Tabala M, Malongo F, Kawende B (2019). HIV viral suppression among pregnant and breastfeeding women in routine care in the Kinshasa province: a baseline evaluation of participants in CQI-PMTCT study. J Int AIDS Soc.

[69] Sandbulte M, Brown M, Wexler C, Maloba M, Gautney B, Goggin K (2020). Maternal viral load monitoring: Coverage and clinical action at 4 Kenyan hospitals. PLoS ONE.

[70] Rosenberg NE, van Lettow M, Tweya H, Kapito-Tembo A, Bourdon CM, Cataldo F (2014). Improving PMTCT uptake and retention services through novel approaches in peer-based family-supported care in the clinic and community: a 3-arm cluster randomized trial (PURE Malawi). J Acquir Immune Defic Syndr.

[71] Sagay AS, Ebonyi AO, Meloni ST, Musa J, Oguche S, Ekwempu CC (2015). Mother-to-Child Transmission Outcomes of HIV-Exposed Infants Followed Up in Jos North-Central Nigeria. Curr HIV Res.

[72] Haas AD, van Oosterhout JJ, Tenthani L, Jahn A, Zwahlen M, Msukwa MT (2017). HIV transmission and retention in care among HIV-exposed children enrolled in Malawi’s prevention of mother-to-child transmission programme. J Int AIDS Soc.

[73] Landes M, van Lettow M, van Oosterhout JJ, Schouten E, Auld A, Kalua T (2021). Early post-partum viremia predicts long-term non-suppression of viral load in HIV-positive women on ART in Malawi: Implications for the elimination of infant transmission. PLoS ONE.

[74] Myer L, Redd AD, Mukonda E, Lynch BA, Phillips TK, Eisenberg A, et al. Antiretroviral adherence, elevated viral load and drug resistant mutations in HIV-infected women initiating treatment in pregnancy: a nested case-control study. Clin Infect Dis. 2020 Jan 16;70(3):501–508. 10.1093/cid/ciz209 PMC7188229 30877752

[75] Onoya D, Sineke T, Brennan AT, Long L, Fox MP (2017). Timing of pregnancy, postpartum risk of virologic failure and loss to follow-up among HIV-positive women. AIDS.

[76] Koss CA, Natureeba P, Plenty A, Luwedde F, Mwesigwa J, Ades V (2014). Risk factors for preterm birth among HIV-infected pregnant Ugandan women randomized to lopinavir/ritonavir- or efavirenz-based antiretroviral therapy. J Acquir Immune Defic Syndr.

[77] Zack RM, Golan J, Aboud S, Msamanga G, Spiegelman D, Fawzi W (2014). Risk Factors for Preterm Birth among HIV-Infected Tanzanian Women: A Prospective Study. Obstet Gynecol Int.

[78] Waithaka D, Kagwanja N, Nzinga J, Tsofa B, Leli H, Mataza C (2020). Prolonged health worker strikes in Kenya- perspectives and experiences of frontline health managers and local communities in Kilifi County. Int J Equity Health.

