# Supplementary material for: Retention in care and viral suppression in the PMTCT continuum at a large referral facility in western Kenya
Source: AIDS Behav. 2022 Apr 25;26(11):3494–505. doi: 10.1007/s10461-022-03666-w (PMC9550706; doi:10.1007/s10461-022-03666-w)
Supplement: Supplementary file 2 — Supplementary Material 2 [file 10461_2022_3666_MOESM2_ESM.docx]

**Supplementary Material**

**Table S1.** Visit characteristics for women during pregnancy and the postpartum period.

| **Characteristic** | **Total**  **n (%)**  **n=856** | **New Positives**  **n (%)**  **n=167** | **Known Positives**  **n (%)**  **n=689** | **Test Statistic** | **P-value** |
| --- | --- | --- | --- | --- | --- |
| # ANC visits, median (IQR) | 1 (0-3) | 1 (0-3) | 1 (0-2) | Z = 4.7 | <0.001 |
| Location of HIV care during pregnancy |  |  |  |  |  |
| HIV clinic | 353 (41) | 37 (22) | 316 (46) | X^2^ = 34.3 | <0.001 |
| ANC | 455 (53) | 113 (68) | 342 (50) |  |  |
| ANC and HIV clinic | 48 (5.6) | 17 (10) | 31 (4.5) |  |  |
| Weeks post-delivery at first PNC visit within 90 days, median (IQR) | 5 (2-6) | 5 (0-6) | 5 (2-6) | LR^a^ = 6.4 | 0.012 |
| # PNC visits post-delivery, median (IQR) | 7 (5-9) | 6 (1-9) | 7 (5-9) | Z = -4.0 | <0.001 |
| 1 day to 12 months | 7.0(5.0,9.0) | 6.0(1.0,9.0) | 7.0(5.0,9.0) | Z = -4.0 | <0.001 |
| 1 day to 18 months | 10 (8-12) | 9 (6-12) | 10 (8-12) | Z = -2.3 | 0.023 |
| Days from attended PNC visit to next scheduled visit, median (IQR) |  |  |  |  |  |
| 1 day to 6 months | 30 (28-56) | 31 (28-55) | 30 (28-56) | Z = 0.3 | 0.791 |
| 7-12 months | 35 (28-61) | 35 (28-62) | 35 (28-61) | Z = 0.9 | 0.343 |
| 13-18 months | 56 (28-81) | 56 (29-77) | 56 (28-82) | Z = 0.2 | 0.837 |

^a^ LR is Log Rank Test

**Table S2.** Characteristics of infants.

| **Characteristics** | **6 weeks**  **n (%) n=746** | **6 months**  **n (%) n=741** | **12 months**  **n (%) n=703** | **18 months**  **n (%) n=658** |
| --- | --- | --- | --- | --- |
| HIV test result |  |  |  |  |
| Positive | 3 (0.4) | 5 (0.7) | 5 (0.7) | 0 (0) |
| Negative | 391 (52) | 253 (34) | 415 (59) | 396(60) |
| Missing | 352 (47) | 483(65) | 283 (40) | 262(40) |
| HIV test type^a^ |  |  |  |  |
| ELISA | 0 (0) | 91 (12) | 230 (33) | 380 (58) |
| DNA PCR | 394 (53) | 167 (23) | 190 (27) | 16 (2.4) |
| Stopped breastfeeding | 11 (1.5) | 95 (13) | 425 (61) | 628 (95) |
| Missing HIV test after stopping breastfeeding^b^ | 6 (0.8) | 94 (13) | 278 (40) | 259 (39) |

^a^ Proportions are based on the number of infants with positive or negative test results.

^b^ Proportions are based on the number of infants that have stopped breastfeeding at each timepoint.

**Table S3.** Factors associated with viral suppression during pregnancy among KHP women at ANC enrollment.

| **Characteristics** | **Unadjusted OR (95% CI)**  **n=396^a^** | **Adjusted OR (95% CI)**  **n=396^a^** |
| --- | --- | --- |
| Age (years) | 1.08 (1.03-1.14) | 1.10 (1.03-1.16) |
| Gestational age (weeks) | 0.98 (0.95-1.02) | 0.98 (0.95-1.02) |
| VL suppressed prior to pregnancy | 4.12 (1.93-8.80) | 4.05 (1.85-8.84) |
| Maximum WHO stage prior to ART initiation |  |  |
| WHO stage 1 or 2 | Ref. | Ref. |
| WHO stage 3 or 4 | 0.53 (0.28-1.01) | 0.45 (0.22-0.90) |
| ART base class |  |  |
| NNRTI | Ref. | Ref. |
| PI/other | 0.68 (0.31-1.50) | 1.08 (0.44-2.64) |
| Time (weeks) on ART | 0.99 (0.99-1.01) | 0.99 (0.99-1.00) |

^a^ Includes 351 virally suppressed and 45 not virally suppressed women.

**Table S4.** Summary statistics by event in the competing risk model.

| **Characteristic** | **Virally suppressed**  **n (%)**  **n=659** | **Death/LTFU n (%)**  **n=51** | **Censored n (%)**  **n=60** |
| --- | --- | --- | --- |
| Age, median years (IQR) | 32 (28-37) | 30 (25-34) | 33 (28-37) |
| Gestational age, median weeks (IQR) | 20 (14-27) | 24 (17-31) | 24 (15-28) |
| WHO stage |  |  |  |
| WHO stage 1 or 2 | 518 (79) | 42 (82) | 47 (78) |
| WHO stage 3 or 4 | 141 (21) | 9 (18) | 13 (22) |
| ART base class |  |  |  |
| NNRTI | 594 (90) | 47 (92) | 52 (87) |
| PI | 65 (10) | 4 (8) | 8 (13) |
| NHP status |  |  |  |
| No | 560 (85) | 34 (67) | 47 (78) |
| Yes | 99 (15) | 17 (33) | 13 (22) |

**Table S5.** Sensitivity analysis of competing risk model for factors associated with time to viral suppression following delivery, including only subjects with ≥1 viral load available in the postpartum period (n=770 with complete data).

|  | **Model of viral suppression** | | **Model of Death/LTFU** | |
| --- | --- | --- | --- | --- |
| **Characteristics** | **Unadjusted OR**  **(95% CI)** | **Adjusted OR**  **(95% CI)** | **Unadjusted OR**  **(95% CI)** | **Adjusted OR**  **(95% CI)** |
| Age (years) | 1.13 (1.11-1.15) | 1.19 (1.13-1.25) | 0.88 (0.86-0.90) | 0.85 (0.80-0.90) |
| Gestational age (weeks) | 1.11 (1.07-1.15) | 1.04 (0.99-1.08) | 0.92 (0.89-0.96) | 0.94 (0.89-0.99) |
| Maximum WHO stage prior to ART initiation |  |  |  |  |
| WHO stage 1 or 2 | Ref. | Ref. | Ref. | Ref. |
| WHO stage 3 or 4 | 2.38 (1.68-3.39) | 0.95 (0.75-1.19) | 1.08 (0.64-1.83) | 1.38 (0.74-2.55) |
| ART base class |  |  |  |  |
| NNRTI | Ref. | Ref. | Ref. | Ref. |
| PI/other | 2.10 (1.56-2.84) | 1.01 (0.79-1.30) | 1.72 (1.12-2.63) | 1.28 (0.69-2.36) |
| NHP status | 2.10 (1.53-2.88) | 1.10 (0.86-1.40) | 1.31 (0.85-2.03) | 1.61 (0.86-3.00) |
